# Supplementary material for: Enriching Human Interactome with Functional Mutations to Detect High-Impact Network Modules Underlying Complex Diseases
Source: Genes (Basel). 2019 Nov 15;10(11):933. doi: 10.3390/genes10110933 (PMC6895925; doi:10.3390/genes10110933)
Supplement: Supplementary file 1 [file genes-10-00933-s001.zip › Supplementary Materials_MDPI-Genes.docx]

**Enriching the human interactome with functional mutations to detect high-impact network modules underlying complex diseases**

**Hongzhu Cui^1^*, Suhas Srinivasan^2^ and Dmitry Korkin^1, 2, 3^***

^1^ Bioinformatics and Computational Biology Program, Worcester Polytechnic Institute, Worcester, MA, USA

^2^ Data Science Program, Worcester Polytechnic Institute, Worcester, MA, USA

^3^ Computer Science Department, Worcester Polytechnic Institute, Worcester, MA, USA

***** Correspondence: [hcui2@wpi.edu](mailto:hcui2@wpi.edu) (H.C.); [dkorkin@wpi.edu](mailto:dkorkin@wpi.edu) (D.K.)

**Supplementary Information**

**List of figures:**

| **Supplementary Figure 1** | Structure-based prediction of SNP’s effect on PPI when applying SNP-IN tool. |
| --- | --- |
| **Supplementary Figure 2** | Comparison of DIMSUM against GWAS based and naïve network propagation procedure. |
| **Supplementary Figure 3** | Coronary artery disease (CAD) module discovered by the SCA algorithm. |

**List of tables:**

| **Supplementary Table 1** | Description of the eight GWAS datasets curated for this work. |
| --- | --- |
| **Supplementary Table 2** | Seeds generated by the Pascal tool from eight GWAS datasets of complex diseases. |
| **Supplementary Table 3** | SNP-IN tool annotation results for eight GWAS datasets. |
| **Supplementary Table 4** | Disease gene association data curated from OMIM, HGMD |
| **Supplementary Table 5** | Overlapping genes between the discovered modules from all three methods |
| **Supplementary Table 6** | Total number of enriched GO terms for eight GWAS datasets from all three methods |
| **Supplementary Table 7** | HIST1H4A-HIST1H3A centered PPI subnetwork and associated disruptive mutations |


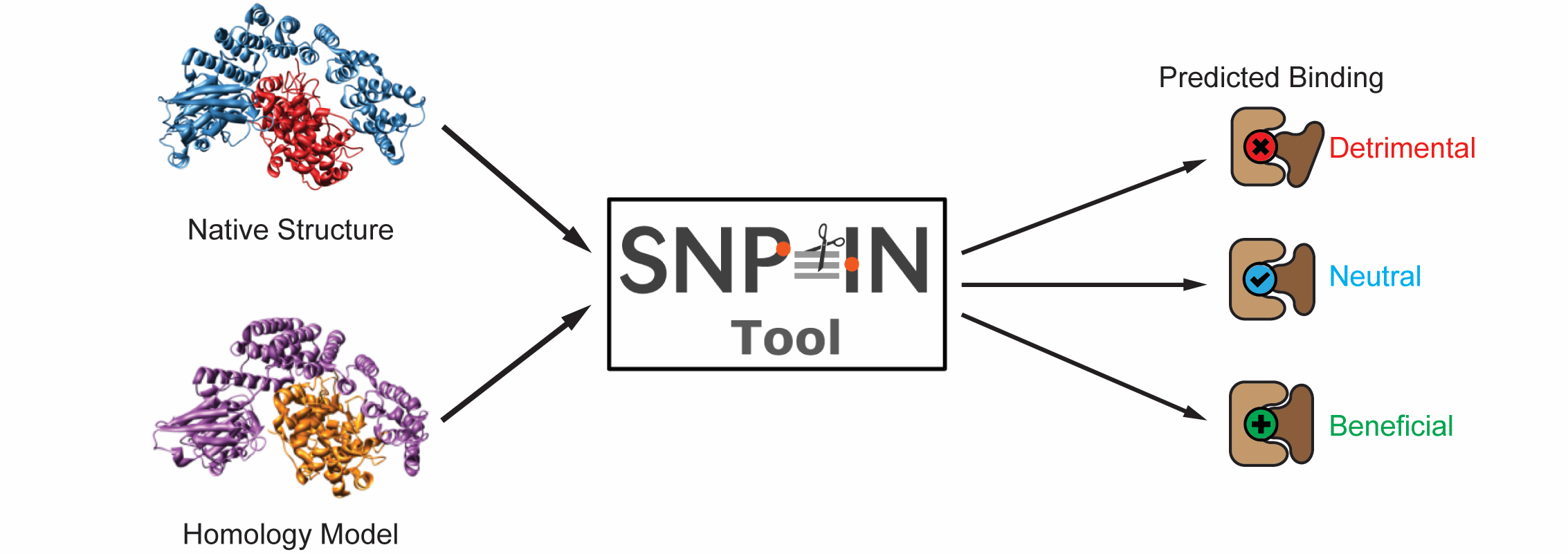


**Supplementary Figure 1.** **Structure-based prediction of SNP’s effect on PPI when applying SNP-IN tool.** The SNP-IN tool uses native structure (when available) or a homology model of the PPI as an input. The output is the predicted PPI-rewiring effect of the SNP with three possible outcomes: (i) Detrimental: where the binding is lost; (ii) Neutral: the binding is preserved and (iii) Beneficial: the binding is strengthened.


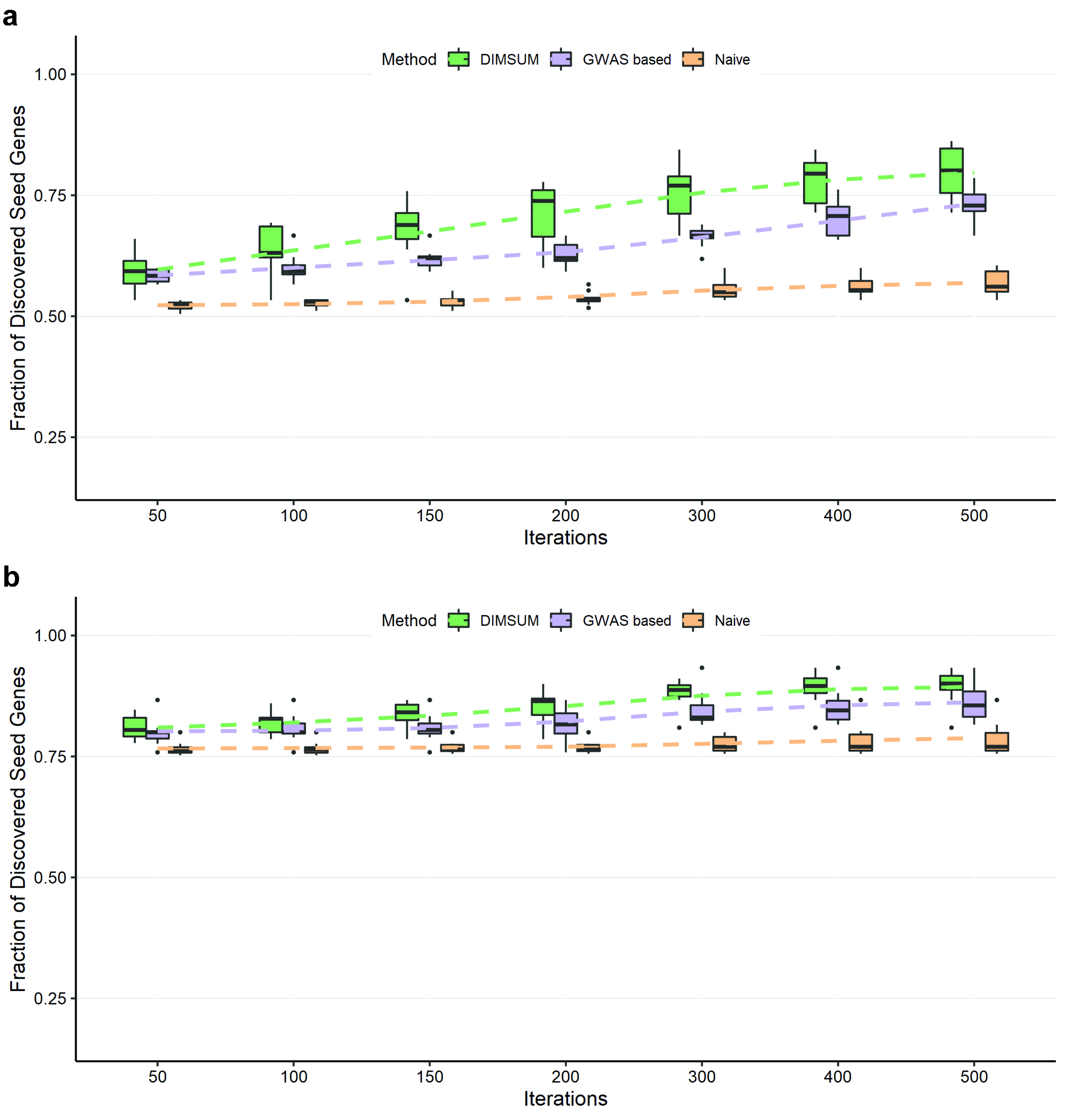


**Supplementary Figure 2.** **Comparison of DIMSUM against GWAS based and naïve network propagation procedure.** (**a**) 50% of nodes are randomly selected from the seed gene pool where DIMSUM has a greater trend in discovering seed genes with respect to the increasing number of iterations reaching an average of 0.75. (**b**) 75% of nodes are randomly selected from the seed gene pool where DIMSUM again outperforms the GWAS based and naïve method, reaching an average discovery rate of 0.9 with 500 iterations.


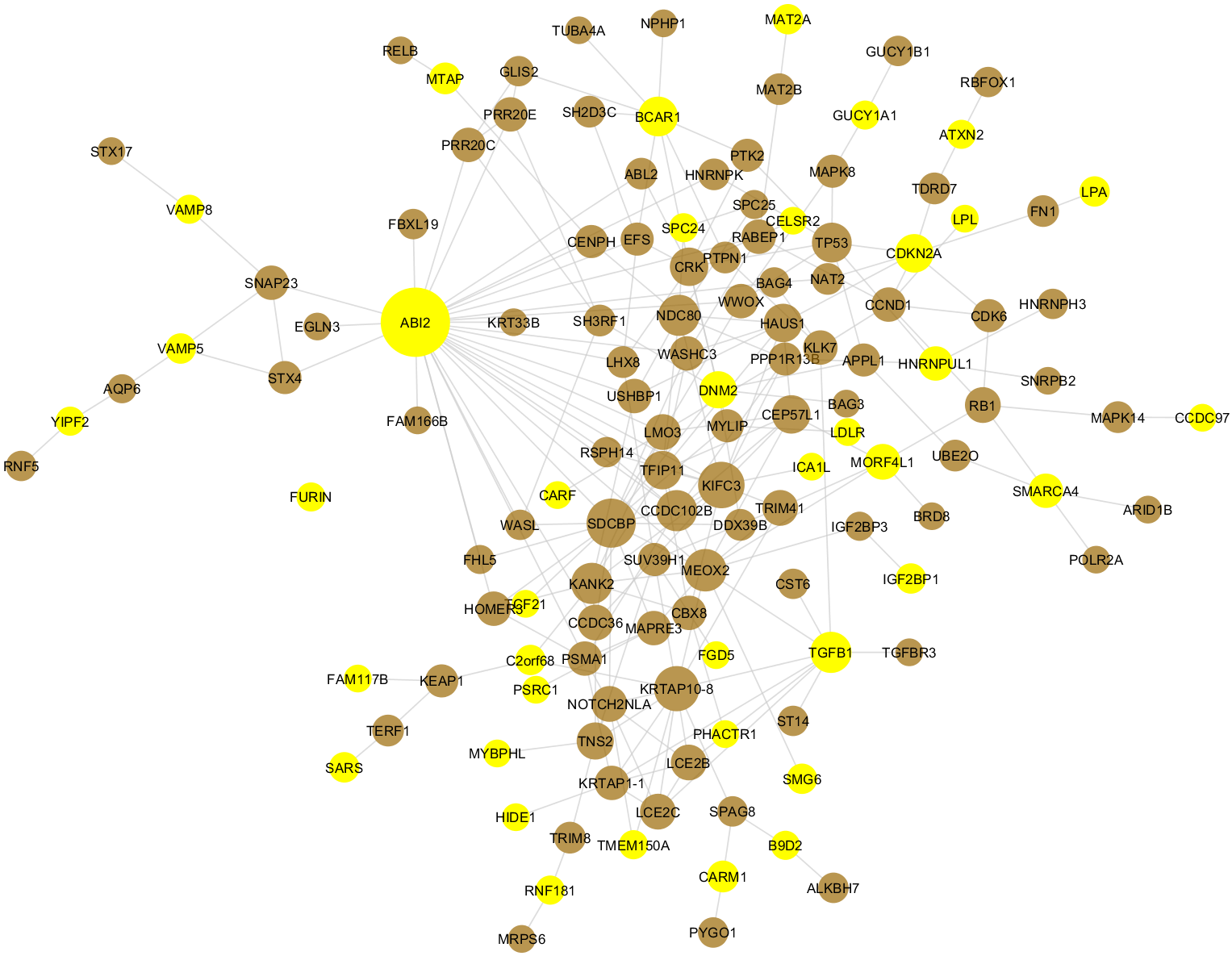


**Supplementary Figure 3.** **Coronary artery disease (CAD) module discovered by the SCA algorithm.** Yellow nodes represent the seed genes for CAD and brown nodes represent the added genes. The SCA algorithm tries to include as many seeds as possible when building the module. No CAD associated genes were discovered by SCA, and the degree distribution was greater (i.e. high degree nodes or hubs were added) than in the module discovered by DIMSUM (Fig. 4).
