# Supplementary figures and images for: Enriching Human Interactome with Functional Mutations to Detect High-Impact Network Modules Underlying Complex Diseases
Source: Genes (Basel). 2019 Nov 15;10(11):933. doi: 10.3390/genes10110933 (PMC6895925; doi:10.3390/genes10110933)

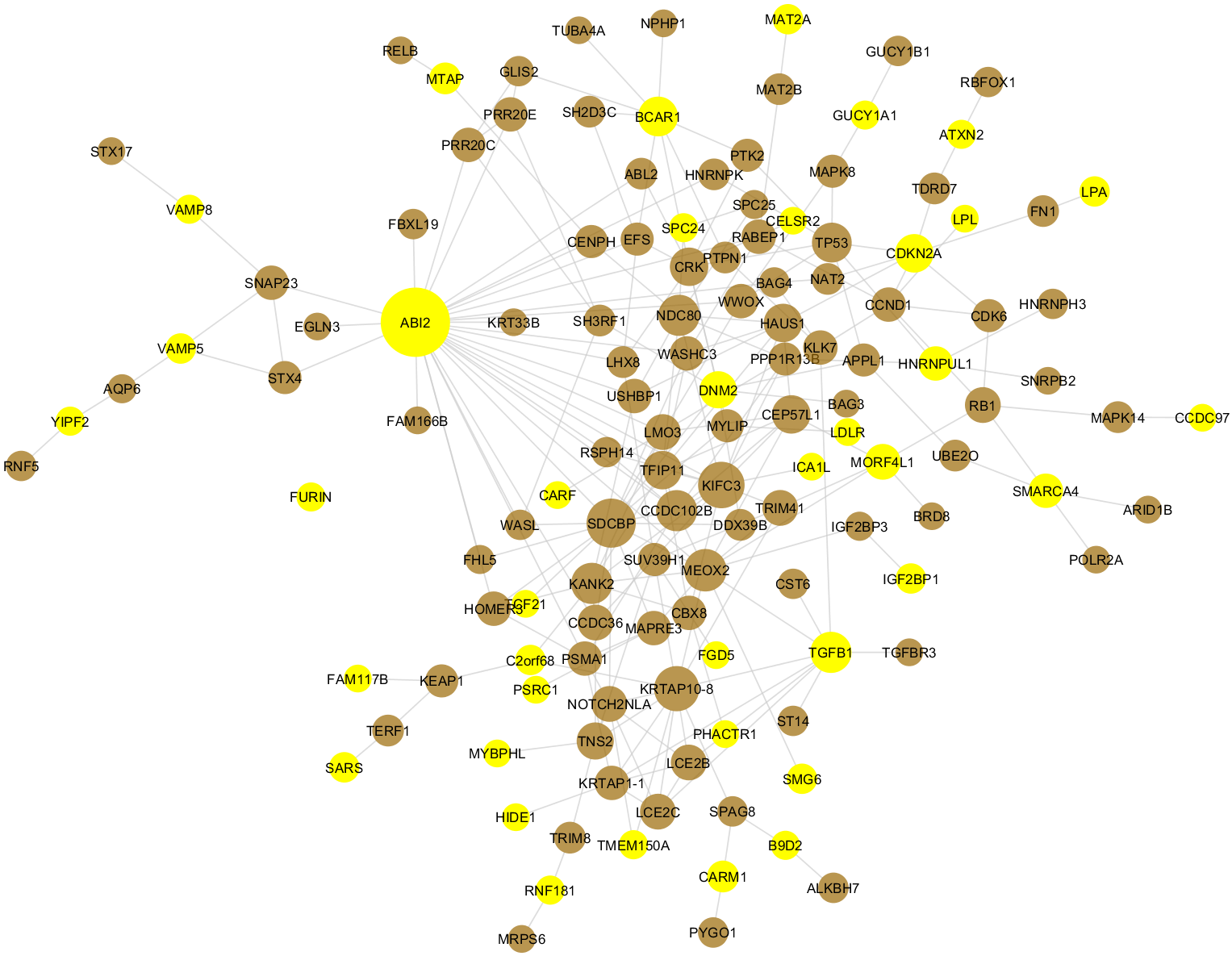

Supplement: Supplementary file 1 [file genes-10-00933-s001.zip › Supp_Fig3.png]

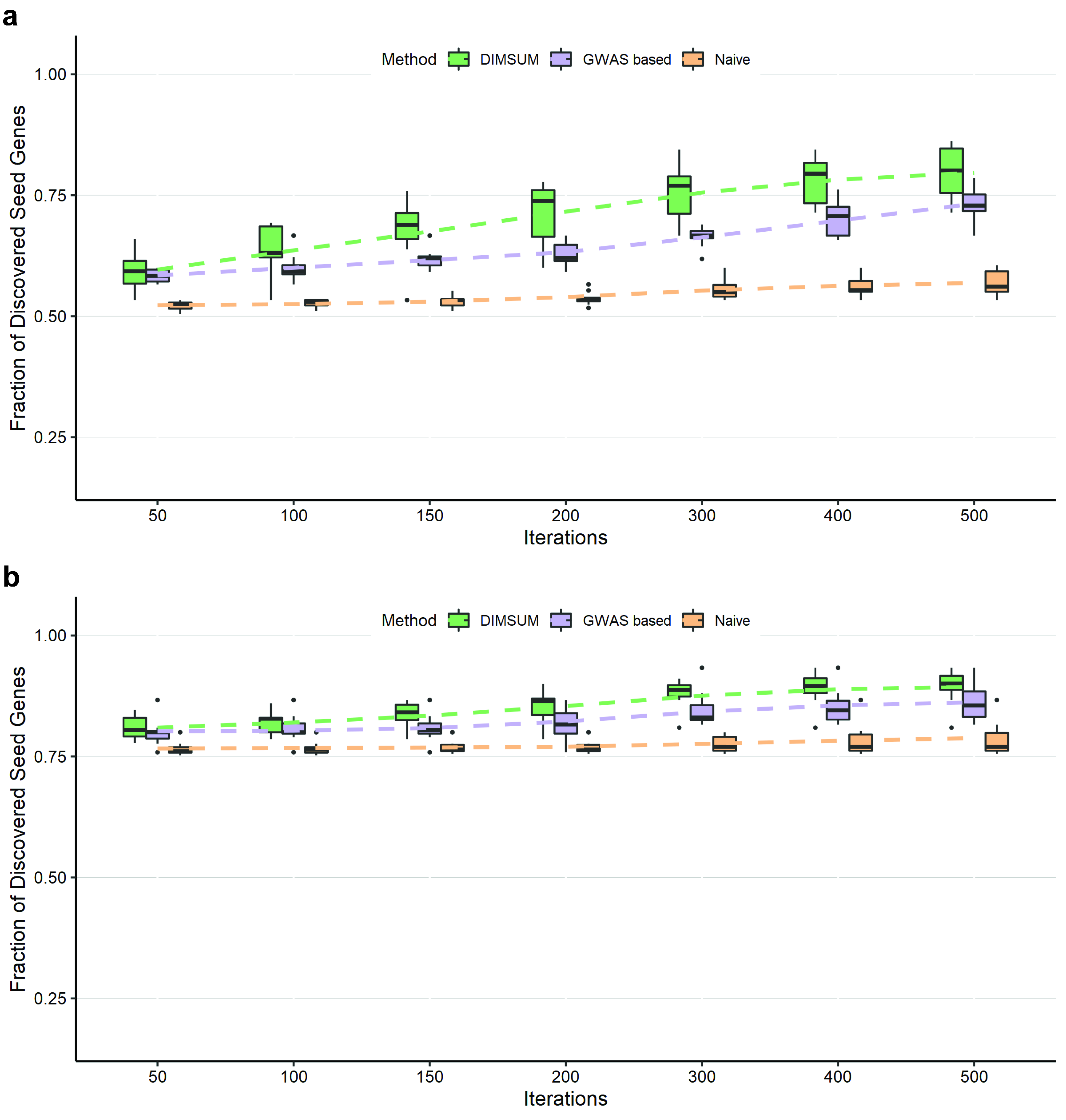

Supplement: Supplementary file 1 [file genes-10-00933-s001.zip › Supp_Fig2.tif]

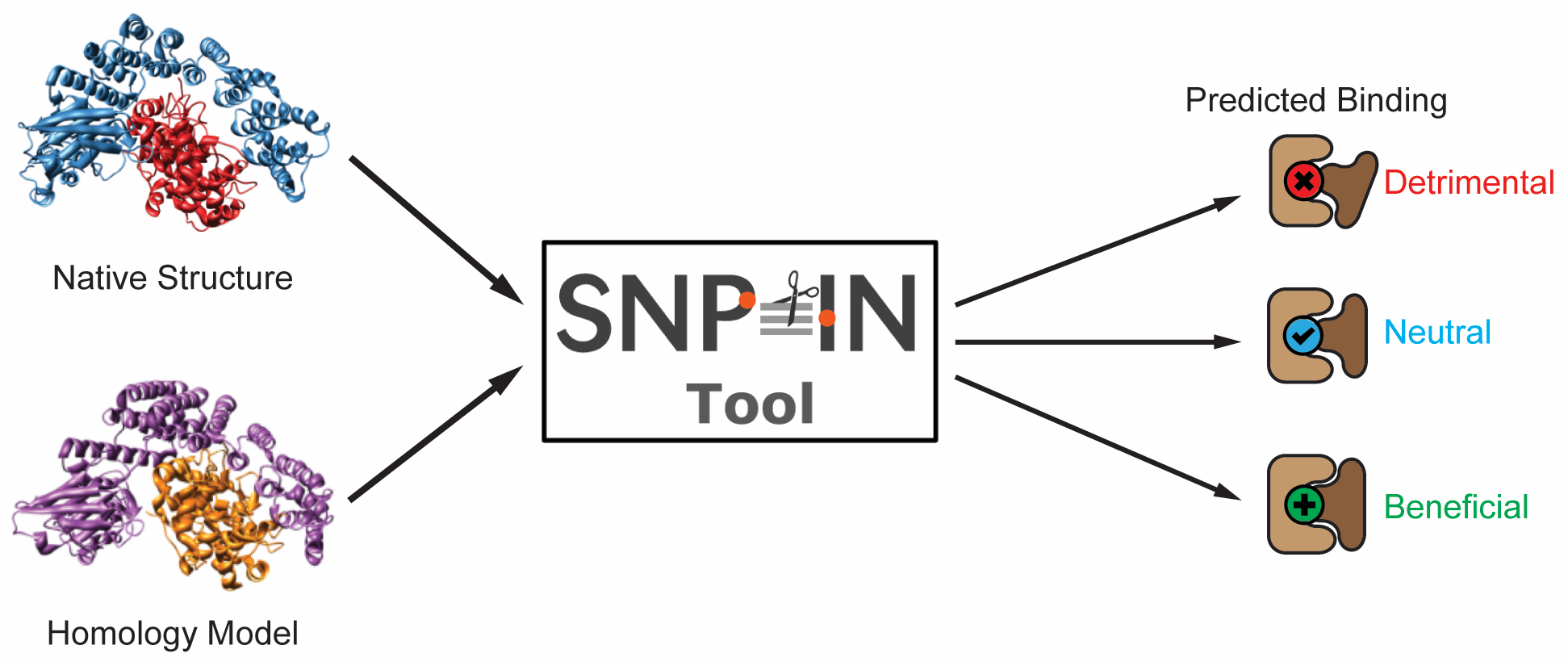

Supplement: Supplementary file 1 [file genes-10-00933-s001.zip › Supp_Fig1.tif]
